# Supplementary material for: Complexome profiling of the Chlamydomonas psb28 mutant reveals TEF5 as an early PSII assembly factor
Source: Plant Cell. 2025 Mar 17;37(6):koaf055. doi: 10.1093/plcell/koaf055 (PMC12164590; doi:10.1093/plcell/koaf055)
Supplement: koaf055_Supplementary_Data [file koaf055_supplementary_data.zip › psb28_interactive.zip!/plots/intensity/qualityPlots/peptides/_Cre06.g261750.t1.2.html]

### Cre06.g261750.t1.2

Trivial name:   
SeqCoverage [median]: 6.90  
Mass: 71.13 kDa  
  
 

| Sequence | MissedCleavage | Mass [Da] | wtIntensity | muIntensity | Score |
| --- | --- | --- | --- | --- | --- |
| AISVLSELTR | 0 | 1087.62 | 0.00 | 989.76 | 105.57 |
| SVSPQPDVAK | 0 | 1026.53 | 282.13 | 0.00 | 59.24 |
| TLGSLFTNVR | 0 | 1106.61 | 791.56 | 705.32 | 78.33 |
| TPIPVSYTR | 0 | 1032.56 | 0.00 | 385.63 | 50.61 |
|  |  |  | 1073.7 | 2080.7 |  |
